# Kampo Medicines for Frailty in Locomotor Disease

**DOI:** 10.3389/fnut.2018.00031

**Published:** 2018-04-26

**Authors:** Hajime Nakae, Yuko Hiroshima, Miwa Hebiguchi

**Affiliations:** ^1^Department of Emergency and Critical Care Medicine, Akita University Graduate School of Medicine, Akita, Japan; ^2^Department of Traditional Japanese Medicine, Akita University Hospital, Akita, Japan

**Keywords:** traditional Japanese medicine, sarcopenia, locomotive syndrome, hypofunctional constitution, trauma, Gosyajinkigan, Jidabokuippo

## Abstract

Frailty is a syndrome that includes broad problems of senility and consists of three domains: physical, psychological, and social. Kampo medicine is used for intervention in cases of hypofunction in a mental or physical state. Kampo treatment, using Hojin formulations such as Hachimijiogan and Gosyajinkigan, is useful in patients with “jinkyo,” or kidney hypofunction. Ketsu includes both blood and its metabolic products that circulate throughout the body. Oketsu is a disturbance of ketsu and is considered to be a microcirculation disorder. Anti-oketsu formulations, such as Keishibukuryogan and Jidabokuippo, are useful in the treatment of trauma patients who are experiencing swelling and pain. “Ki” is the universal energy that exists in the world. Hoki formulations, such as Rikkunshito and Hochuekkito, are useful in patients with poor appetites for reinforcing vital energy. Juzentaihoto and Ninjinyoeito are useful in patients with hypofunction of ki and ketsu, which are accompanying symptoms of coldness or cutaneous dryness. Thus, Kampo medicines can be used as a superior approach for the management of frailty.

## Introduction

Recently, sarcopenia, frailty, and locomotive syndrome have become known as disorders that are mainly caused by aging (1). These disorders have been considered in the context of a long life expectancy and illustrate the importance of promoting preventive care in Japan where the aging society progress (2, 3).

Here, we provide an outline regarding prevention and intervention for frailty in locomotor disease using Kampo medicine.

## Frailty and sarcopenia

Since physiological residual function decreases in a senile state, it becomes difficult to endure a higher level of stress, thereby resulting in frailty. Notably, frailty is considered to be a state that is easy to enter into, in the context of a bionomics disorder, and may require nursing care.

Sarcopenia is a syndrome that is characterized by progressive and generalized loss of skeletal muscle mass and strength, with a risk of adverse outcomes such as physical disability, poor quality of life, and death (4). Further, frailty includes both physical and mental problems, such as cognitive dysfunction or depression, and social problems, including economic hardship (5). Thus, frailty is a problem that is widely indicative of a senile state, consisting of these three domains. Frailty is defined as a clinical syndrome in which three or more of the following criteria are present: unintentional weight loss (10 lbs. in the previous year), self-reported exhaustion, weakness (grip strength), slow walking speed, and low physical activity (6). Among these criteria, weakness and slowness are also indicative of sarcopenia, suggesting that sarcopenia is a central element of a physical frailty (7).

## Intervention and prevention of frailty

For intervention and prevention of frailty, exercise therapy and nutrition care for sarcopenia is critical. Elderly people cannot perform exercise therapy because of weakening physical strength and a variety of physical symptoms. Pain control plays a key role in locomotor disease. However, elderly people cannot often utilize nonsteroidal anti-inflammatory drugs (NSAIDs) because of gastrointestinal (GI) disorder or renal dysfunction. Furthermore, it may be difficult to tolerate Western medicines, due to the side effects of anticonvulsant or opioid drugs.

**Figure 1:**
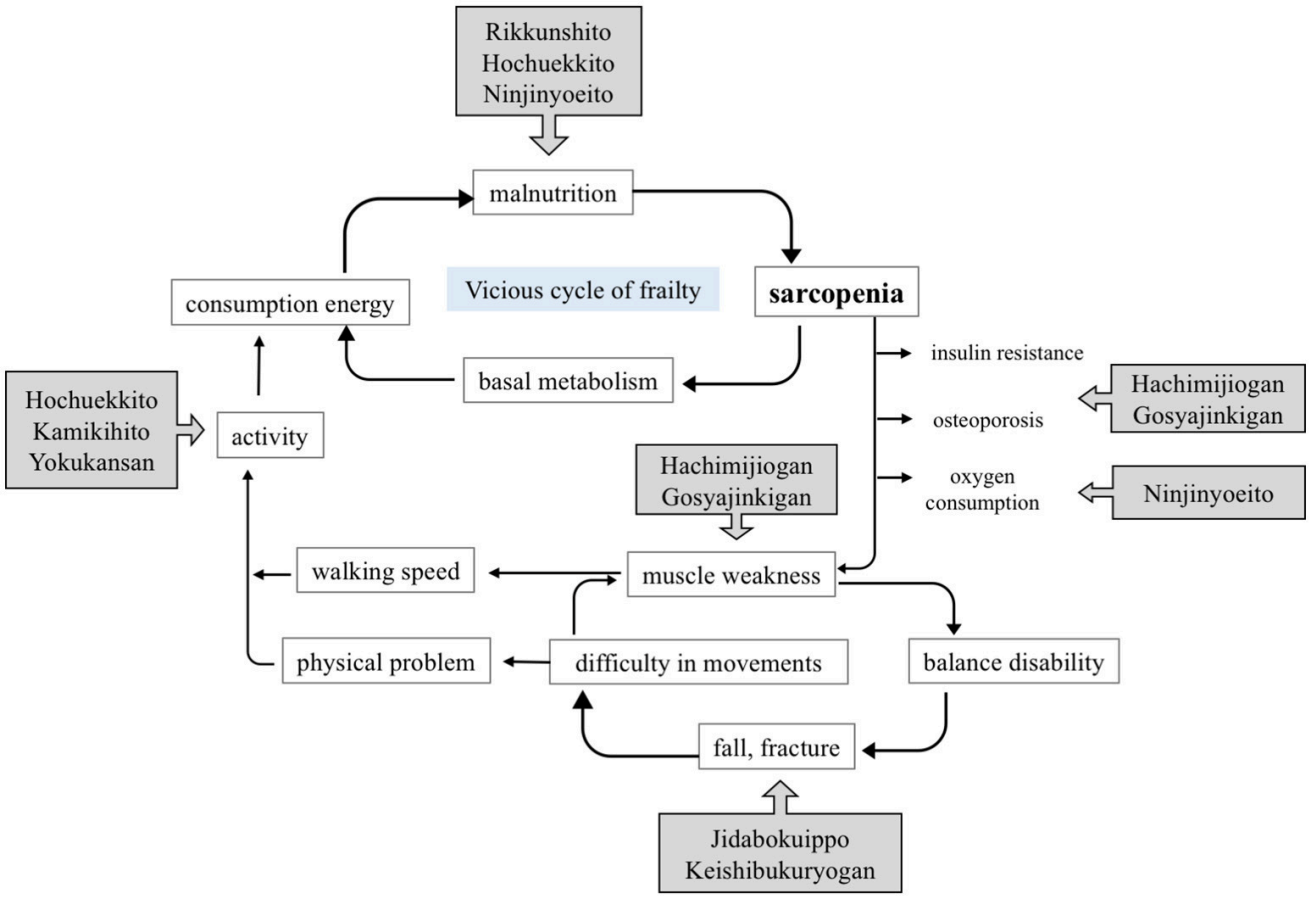
Approach to frailty treatment, using Kampo medicines.

In such cases, Kampo medicines may be useful to raise of physical strength and improve appetite (Figure 1).

## Treatment with kampo medicine for the locomotor disease

Frailty is a state between disability and robust health. If appropriate intervention is performed against frailty, it is possible for frail patients to return to a healthy state (8). This concept is similar to “mibyo,” or presymptomatic disease, in Kampo medicine. Mibyo is a state between illness and health, which can require treatment to prevent diseases from worsening and spreading to other parts of the body. Western medicine has no tests and, hence, no treatments available for people who have minor symptoms; however, Kampo recognizes even the slightest of these abnormalities, and takes action against them. In short, intervention by Kampo preparations and lifestyle guidance can change the body, such that it does not become ill, thereby preserving the health of the individual.

Frailty includes mental problems. In Kampo medicine, since ki, the universal energy that exists in the world, is considered to be a basic element of life, it is thought to support all mental, as well as physical, functions and bodily structures. This concept is referred to as the “relation of mind and body.” Therefore, there are similarities between the philosophy of Kampo medicine and frailty, such that mind and body are regarded as inseparable.

Kampo medicine intervenes in cases of hypofunction in a mental or physical state, in a manner that is known as hypofunctional constitution. Notably, when elderly people with coldness suffer from reduced lower limb and bladder function, Kampo treatment is well applied. These symptoms are diagnosed as “jinkyo,” kidney hypofunction. Hojin formulations for jinkyo, such as Rokumigan (RG), Hachimijiogan (HJG), and Gosyajinkigan (GJG), are used. HJG consists of Rehmannia Root, Cornus Fruit, Dioscorea Rhizome, Alisma Rhizome, Hoelen, Moutan Bark, Cinnamon Bark, and Aconite Tuber. It is applied in cases of nephritis, diabetes, impotence, sciatica, lumbago, bladder catarrh, prostatic hypertrophy, and hypertension, among others. Kawago has studied whether HJG can improve quality of life in Japanese patients with peripheral arterial disease (PAD). In the patients with the intermittent claudication caused by PAD, it was suggested that HJG administration might improve quality of life (10). GJG is a prescription that adds Achyranthes Root and Plantago Seed to HJG. It is applied in cases of lumbago, coldness of the lower part of the body, micturition abnormalities (diuresis, urinary frequency, night urination), visual impairment (cataracts, blurred vision), hearing impairment (hearing loss, tinnitus), and edematous tendency. GJG is typically used for numbness in the lower limbs. In terms of pain relief effects, central analgesia has been observed through the stimulation of the κ opioid receptor, whereas peripheral analgesia has been observed through increased nitric oxide-production (11, 12). In a mouse model of neuropathic pain, analgesia has been reported through suppression of TNF—α expression from activated microglia (13). In a clinical setting, it was reported that GJG contributed to the suppression of peripheral neuropathy with the FOLFOX therapy for colorectal cancer (14). However, GJG did not prevent oxaliplatin-associated peripheral neuropathy in a multicenter randomized phase III trial (15). There remains controversy regarding whether GJG prevents chemotherapy-induced peripheral neuropathy in patients undergoing neurotoxic chemotherapy (9). Watanabe investigated whether GJG reduces the onset of diabetic complications (16). After 5 years of observation, 116 patients underwent analysis. Deterioration of the ankle reflex was suppressed in the GJG group. In addition, glycated hemoglobin and fasting plasma glucose were reduced in the GJG group. Kishida showed that GJG suppressed sarcopenia via the IGF-1/insulin pathway, maintained the expression of mitochondrial-related transcription factors, and suppressed TNF-α in senescence-accelerated mice (17). These results indicate that GJG is a promising candidate for relief from sarcopenia. Satoh reported that decreased pharmacological aging was observed in a single medical preparation, and that multicomponent composition medicines, such as Hojin formulations, showed an anti-aging effect (18). Interestingly, the vascular relaxant effect differs among three preparations (RG, HJG, and GJG; Tables 1, 2). Thus, GJG improves disuse atrophy through analgesia and prevents frailty. Herbal formulations containing Rehmannia Root as major ingredient are known as Rehmannia drug group. Since the Rehmannia drug group does not include licorice, there is no risk of pseudoaldosteronism. Nevertheless, Rehmannia Root might cause a GI disorder. When GI disorder develops, the administration of the Rehmannia drug group should be avoided before a meal; rather, it should be taken after a meal. Another option is the use of Rehmannia Root together with Anchusan (AS); this combination is used in patients with hypofunctional constitution and abdominal pain, as well as heartburn, nausea, vomiting, epigastric discomfort, and malaise. Aconite Tuber is also added to improve “ki,” which gradually decreases through aging. Aconite Tuber provides antioxidant activity, analgesia, and an increased vascular flow (19, 20). Small amounts of Aconite Tuber can be used for arthralgia and somatic pain with coldness in elderly patients (21). Even in a kidney hypofunction state (jinkyo), when the clockwise circulation of ki is disturbed or a GI disorder is caused by Rehmannia Root, the Keishito (KT) group should be used instead. KT is used in patients who exhibit signs such as hypofunction, poor digestion, and exhaustion. KT consists of five herbs; Cinnamon Bark, Peony Root, Jujube Fruit, Ginger Rhizome, and Glycyrrhiza Root. Each formulation in KT group has a modified admixture based on the combination of the five KT herbs. Keishikajutsubuto (KKJBT) is a prescription that includes additional Atractyloids Lanceae Rhizome and Aconite Tuber, in combination with KT. It is applied in cases of coldness or to manage symptoms that become worse in cold (22). Kakkonkajutsubuto (KTKJBT) is a prescription that includes additional Atractyloids Lanceae Rhizome and Aconite Tuber, in combination with Kakkonto. It is applied in cases of pain and stiffness of the upper body (23).

**Table 1:** Vasodilatory activities in Hojin formulations ((9), revised).

	**Pharmacological action**	**Rokumigan**	**Hachimijiogan**	**Gosyajinkigan**
Vascular endothelial cell	Nitric oxide synthesis	+	+	+
	Prostaglandin I_2_ synthesis	−	−	−
Vascular smooth muscle cell	Calcium channel inhibitor	+	+	+
	Protein kinase C inhibitor	+	+	+
	Beta receptor agonist	−	+	+

**Table 2:** Behaviors in Hojin formulations ((9), revised).

Pharmacological action	Clinical effect
Calcium channel inhibitor	Anti-atherogenic effect
Beta receptor agonist	Improvement of urinary disturbance
Protein kinase C inhibitor	Improvement of coldness in legs
Anticoagulant and antithrombogenic activities	Improvement of numbness in legs
Superoxide dismutase mimicking activity	
Lipid metabolism regulation	
Glycometabolism regulation	
Diuretic activity	
Improvement of kidney function	

**Figure 2:**
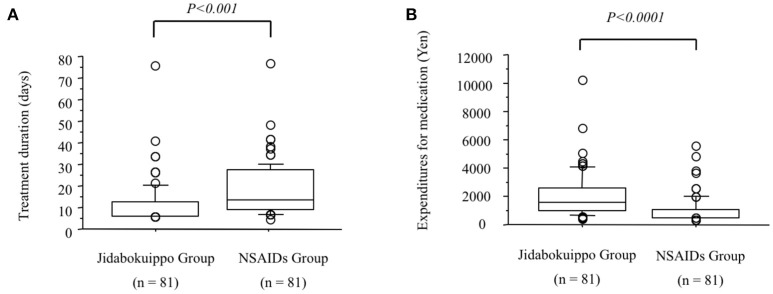
Comparison of treatment durations and expenditures for medication between the jidabokuippo (JDI) and the non-steroidal anti-inflammatory drugs (NSAIDs) groups ((24) revised). **(A)**. Median treatment duration is significantly lower in the JDI group than in the NSAIDs group (*P* < 0.001). **(B)** Median expenditures for medication are significantly lower in the JDI group than in the NSAIDs group (*P* < 0.0001).

**Figure 3:**
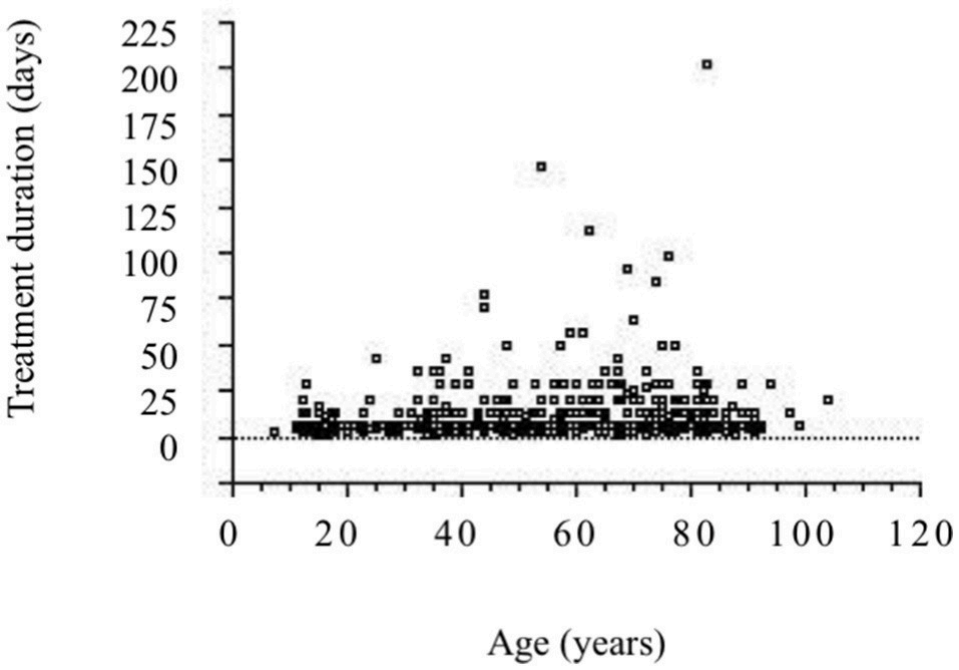
Relationship between ages and treatment durations ((25) revised). A significant correlation is observed between the parameters. (*n* = 643, *r* = 0.150, *P* < 0.0005).

“Ketsu” means red liquid. Ketsu includes both blood and its metabolic products that circulate throughout the body supplying nutrients to cells under the high-level control of ki. “Sui” means colorless liquids, bodily fluid or water. Sui includes all bodily fluids other than ketsu. Ki is represented by the nervous system, ketsu immunological function, and sui the various hormonal secretions that affect metabolism according to the theory of ki, ketsu, and sui. Swelling and pain caused by trauma is regarded as “oketsu.” Oketsu is a disturbance of ketsu and is considered to be a microcirculation disorder. Keishibukuryogan (KBG), Jidabokuippo (JDI), and Tsudosan (TS) are used to treat oketsu. Kampo formulations exhibit antioxidant activity that is thought to be associated with swelling reduction (26, 27). We compared the efficacies of JDI and NSAIDs in patients with rib fracture by analyzing the treatment duration (24). Our results suggested, that compared with NSAIDs, JDI could shorten the duration of treatment and may provide a promising analgesic agent, from a medical economic viewpoint (Figure 2). It is unclear whether JDI effectively shortens the healing period or whether NSAIDs effectively delay healing. In either case, our results prove the non-inferiority of JDI to NSAIDs. We have used JDI for the treatment of various traumas, including multiple injuries, as well as simple bruise and sprain (28, 29). In our previous study, the efficacy rate was 91.6% in 643 patients with trauma who underwent JDI treatment (25). The necessary treatment period became longer with age (Figure 3). This may reflect the fact that wound healing increases with aging. Early intervention for pain control caused by trauma is necessary for elderly people, for the purpose of frailty prevention.

Even in a case of locomotor disease, when a patient complains of poor appetite, which is caused due to deficiency of ki, Hoki formulations for reinforcing vital energy, such as Rikkunshito (RT) and Hochuekkito (HET), should be used initially. Juzentaihoto (JTT) and Ninjinyoeito (NYT) are used for concomitant hypofunction of ki and ketsu, along with the accompanying symptoms of coldness or cutaneous dryness. NYT demonstrates hematopoietic activity and is effective for osteoporosis management (30). Frailty and apathy negatively affect the progression of Alzheimer's disease (31, 32). Ohsawa reported that NYT was effective for anorexia of aging in Alzheimer's disease (33).

Needless to say, Kampo medicines are not magical panaceas for frailty with locomotor disease. In order to engage in these problems, cooperation among various types of professions is required.

## Conclusion

Pain control, as well as nutritional and mental management, are important for cases of frailty with locomotor disease. Since Kampo formulations are composed of multiple crude drugs, a single prescription can address several symptoms simultaneously. Thus, Kampo medicines can be used as a superior approach for the treatment of frailty.

## Ethics statement

As for our studies performed at Akita University Hospital, informed consent was obtained from all of the patients and their families involved at the time of their enrollment. The study was performed with the approval of the ethic committee of the Akita University Hospital and was performed in accordance with the guidelines of good clinical practice.

## Author contributions

HN conceived the original idea, and developed it in collaboration with YH and MH. HN wrote the first draft of the article. All authors contributed to revisions.

### Conflict of interest statement

HN has received lecture fees from Tsumura & Co. The other authors declare that the research was conducted in the absence of any commercial or financial relationships that could be construed as a potential conflict of interest.
